# Translation, Cross-Cultural Adaptation, and Validation of a Dutch Version of the Actions and Feelings Questionnaire in Autistic and Neurotypical Adults

**DOI:** 10.1007/s10803-021-05082-w

**Published:** 2021-05-18

**Authors:** Hedwig A. van der Meer, Irina Sheftel-Simanova, Cornelis C. Kan, James P. Trujillo

**Affiliations:** 1 Academic Centre for Dentistry Amsterdam (ACTA), University of Amsterdam and Vrije Universiteit (VU) University Amsterdam, Department of Orofacial Pain and Dysfunction, Amsterdam, The Netherlands; 2 Center of Expertise Urban Vitality, Faculty of Health, Amsterdam University of Applied Sciences, Amsterdam, The Netherlands; 3 One Planet Research Centre, Radboud University Medical Centre, Radboudumc, Nijmegen, The Netherlands; 4 Department of Psychiatry, Radboud University Medical Centre, Radboudumc, Nijmegen, The Netherlands; 5 Donders Institute for Brain, Cognition, and Behavior, Nijmegen, The Netherlands; 6 Max Planck Institute for Psycholinguistics, Nijmegen, The Netherlands

**Keywords:** Questionnaire, Translation, Autism, Motor cognition, Action

## Abstract

The actions and feelings questionnaire (AFQ) provides a short, self-report measure of how well someone uses and understands visual communicative signals such as gestures. The objective of this study was to translate and cross-culturally adapt the AFQ into Dutch (AFQ-NL) and validate this new version in neurotypical and autistic populations. Translation and adaptation of the AFQ consisted of forward translation, synthesis, back translation, and expert review. In order to validate the AFQ-NL, we assessed convergent and divergent validity. We additionally assessed internal consistency using Cronbach’s alpha. Validation and reliability outcomes were all satisfactory. The AFQ-NL is a valid adaptation that can be used for both autistic and neurotypical populations in the Netherlands.

**Supplementary Information:**

The online version contains supplementary material available at 10.1007/s10803-021-05082-w.

## Introduction

The hallmark of autism spectrum conditions (ASC), also known as autism spectrum disorders (ASD), is difficulties with social interaction, although atypicalities related to the motor system are also common (Bhat et al., 2011; Rinehart et al., 2002). This can be seen in movement production, where simple movements (Cook et al., 2013), interactive movements directed at tablet computers (Chua et al., 2019), and gestures (redacted., in prep) show different kinematic patterns compared to neurotypical individuals. Perception of human movement may also differ in ASC, as there is some evidence that autistic individuals have difficulty recognizing subtle differences in action kinematics (Di Cesare et al., 2017; Rochat et al., 2013), and are less likely to use one person’s action to predict the action of a conspecific (Chambon et al., 2017; von der Lühe et al., 2016).

Atypicalities in the production and perception of movement are highly relevant for understanding ASC, as movement is itself an important aspect of human social interaction. Through visual signaling, we are able to express internal states (e.g.,mood), signal intentions, and form more complex, multimodal utterances (Holler & Levinson, 2019). During social interaction, we must therefore also be able to interpret the visual signals produced by our interlocutor. The production and comprehension of action (i.e., purposeful human movement) are thought to be functionally connected, a theory known as “motor cognition” (Jackson & Decety, 2004).

Motor cognition is focused largely on the idea that similar components of the motor system are involved in the production of goal-directed actions as well the perception or interpretation of the actions of others. This overlap, or link, between production and perception likely utilizes the fact that our actions are shaped by context and intention (Cavallo et al., 2016; Trujillo et al., 2018). In this way, our intentions are embedded in the way we move (Runeson & Frykholm, 1983). Similarly, our emotional state can influence our facial expressions (Ekman, 1993), body posture (Atkinson et al., 2004), and even our action kinematics (Fourati & Pelachaud, 2015). This means that the way we move is rich with information about our intentions and internal state.

The ability to recognize the intentions of others has been linked to empathic ability (Ciaramidaro et al., 2014; Kaplan & Iacoboni, 2006), suggesting that understanding others’ emotions is linked to the ability to understand the meaning and (non-emotional) intention of an action. Intact motor cognition is therefore important for successful social functioning. That motor cognition seems to work differently in autistic individuals (i.e., those diagnosed with ASC) compared to neurotypical individuals (i.e., those not diagnosed with ASC), has led to the hypothesis that movement differences between neurotypical and autistic individuals directly contribute to difficulties in social interaction (Cook, 2016).

Given the importance of the motor system in social functioning, it is important to be able to quantify the extent to which an individual expresses and is able to read the internal states of others through action or movement. This is particularly relevant for clinical populations such as ASC. The actions and feelings questionnaire (AFQ; Williams et al., 2016) is a short, self-report measure that is designed to capture this link between action and internal states in adolescents and adults. Specifically, it is designed to measure one’s self-awareness of own and other actions, quantifying one’s use of gesture and action imagery and expression in daily life, and specifically during social communication, with higher AFQ scores indicating better performance in these domains. The AFQ has been applied to ASC populations, and is able to differentiate between ASC and neurotypical individuals (Williams & Cameron, 2017). Therefore, this measure provides a simple self-report tool that may help to provide more fine-grained distinctions between ASC individuals, for example by tapping into how autistic individuals themselves perceive their use and of understanding of motor imagery, providing insights into the visual or motoric aspect of empathy, which is crucial for human communication, but may be captured less well by other empathy measures (Fletcher-Watson & Bird, 2020; Huggins et al., 2019). This has implications for clinical screening as well as for researchers trying to account for inter-individual differences.

While the AFQ is a promising tool for capturing self-awareness of actions, gesture use, and action/movement-based understanding of others, and may be particularly useful for researchers and clinicians working with ASC, the questionnaire is currently only available in English. Translation and cross-cultural adaptation of the AFQ not only allows researchers and clinicians in other countries to benefit from the tool, but it also allows the possibility of cross-cultural comparisons using the same measurement tools.

Therefore, the primary aim of this study was to translate and cross-culturally adapt the AFQ into Dutch. The second aim was to establish the face validity, construct validity, and internal consistency of the Dutch AFQ. This study provides the AFQ to a new group of researchers and clinicians, and ensures the validity of the tool.

## Methods

### Participants

Twenty-five autistic individuals (15 female; 23 right-handed) and twenty-five neurotypical individuals (14 female; 21 right-handed) participated in the study. No participants were excluded from analysis. Autistic participants were recruited from the Radboud University Medical Centre (UMC), Nijmegen. Patients were recruited via two routes. In the first route, patients were contacted by their psychiatrist at the Radboud UMC with general, global information about the study and asked if they agree to being approached by researchers. In the second route, a message was posted private, organization specific social network, where Radboud UMC psychiatrists have message board style contact with past patients. All participants were clinically diagnosed with Autism Spectrum Condition according to the criteria defined in the DSM-5 (American Psychiatric Association, 2013). The neurotypical control group was recruited via the Radboud University participant recruitment system (SONA), which allows for pre-signup screening of several participant characteristics. For both groups, potential participants were excluded if they had a history of any other (neuro-)psychiatric disorders, brain surgery or brain trauma, or use of anti-psychotic medication. Participants were required to be proficient in Dutch and have normal or corrected-to-normal vision. By starting recruitment of the ASC group first, we were able to pre-screen our control group in an attempt to match age and gender between the two groups. We additionally collected data on education and handedness for further group matching. The study was approved by a local ethics committee (CMO Arnhem-Nijmegen). All participants provided informed consent regarding the procedure and purpose of the study.

### Translation and Adaptation

The translation and cross-cultural adaptation was done in four steps: (1) independent forward-translation, (2) synthesis, (3) back-translation, and (4) expert check (face validity). This process was based on established guidelines for translation and cross-cultural adaptation of questionnaires (Beaton et al., 2000).

In the *Forward Translation,* two independent, native speakers of Dutch, who were also fluent in English, independently translated the AFQ into Dutch. Neither translator was aware of the specific purpose of the questionnaire. One translator (T1) had no experience in the academia, while the other translator [T2; author CC] is a researcher with experience in clinometric studies.

After this forward-translation phase, the two Dutch translations of the AFQ were analyzed by one of the researchers [author CC] together with one of the translators (T2) in order to come to a consensus on a synthesized version (T12) of the AFQ (*Synthesis*).

*Back-translation* of T12 was performed by a native English speaker, who is also fluent in Dutch, and not involved in this project or familiar with the AFQ. After translating the synthesized Dutch (T12) questionnaire back into English (BT1), an expert in the field of autism research and clinical care [author CC], together with author [redacted], checked if the synthesized Dutch version (T12) adequately reflected the original English version. This final version was also checked by an independent, native Dutch speaker in order to ensure the accessibility and clarity of the language used. This resulted in the final Dutch version of the AFQ (AFQ-NL).

### Validity

To assess content validity, we checked the face validity of the AFQ-NL by ensuring that this adaptation reflected the construct that the tool is meant to measure (Mokkink et al., 2010, 2020). This was done by an expert clinician in the field of ASC [author CC], who compared the back-translation (Dutch to English) of the AFQ-NL to the original English version and assessed with the back-translation was comparable in content to the original AFQ, and that the Dutch version accurately reflected the same constructs as the original and back-translated English version.

To assess construct validity, we tested whether the AFQ total score correlates with autism quotient (AQ) (Hoekstra et al., 2008) score. This test of convergent validity is based on Huggins et al. (2019) finding that AFQ negatively correlates with broad autism phenotypes in neurotypical adults (Huggins et al., 2019), and the suggestion that difficulties in recognizing emotions in oneself and in others are common in ASC (Bird & Cook, 2013; Kinnaird et al., 2019). This was tested using a linear model, with AFQ as dependent variable and AQ as independent variable. As a test of divergent validity, we similarly tested for a correlation between total AFQ score and IQ score, as estimated by the WASI-IV short form (Wechsler, 2011). This was done as there are no indications that the AFQ should be correlated with general intelligence. All tests were carried out in the statistical program R (R Core Team, 2013).

### Reliability: Internal Consistency

The internal consistency of the AFQ-NL was assessed in order to ensure that this adapted version also provides consistent responses. Utilizing the R package *psych* (Revelle, 2020)*,* we calculated Cronbach’s alpha to assess overall consistency (total scale), as well as for each of the subscales described in (Williams & Cameron, 2017). In short, these subscales emerged based on confirmatory factor analysis, and consisted of three subscales: Feelings, Imagery, and Animation. Adequate consistency is considered to be an alpha between 0.7 and 0.9 (Nunnally & Bernstein, 1994).

## Results

Participant characteristics, including age, AQ, and AFQ score are provided in Table 1. One of the participants did not identify as male or female, and is thus included in the pooled sample statistics, but not given a separate column in the Table due to privacy reasons.

**Table 1:** Participant characteristics

	Pooled sample	Group comparison (t)	Male	Female	ASC total	Male	Female	NT total	Male	Female
Age (SD)	26.327 (4.60)	3.253*	25.600 (4.82)	26.643 (4.69)	28.455 (4.97)	26.750 (6.14)	29.308 (4.25)	24.240 (3.72)	24.545 (3.86)	24.000 (3.742)
Gender (%)		χ^2^ = 1.871	21 (42%)	28 (56%)		10 (40%)	14 (56%)		11 (44%)	14 (56%)
AQ (SD)	23.840 (10.69)	7.650**	22.526 (7.81)	24.115 (11.89)	30.609 (7.32)	28.667 (4.87)	32.462 (8.49)	15.095 (6.12)	16.111 (4.91)	14.333 (7.01)
AFQ (SD)	27.280 (6.73)	4.961**	24.737 (52.21)	26.578 (7.44)	23.783(5.99)	22.222 (3.42)	24.615 (7.26)	32.095 (5.11)	30.000 (4.21)	33.667 (5.31)
IQ (SD)	111.90 (17.44)	0.124	114.22 (18.17)	109.63 (16.10)	110.81 (22.59)	114.06 (25.31)	107.19 (21.27)	112.98 (10.39)	114.37 (7.23)	111.90 (12.49)

*ASC* autism spectrum conditions, *NT* neurotypical, *SD* standard deviation

*p < 0.05

**p < 0.001

### Translation and Adaptation

During the expert comparison of the synthesized (T12), back-translation, and original version, one small adjustment was made to the wording. In Question 9, the word “normale” (*“normal”)* was changed to “gewone” (*“common”*), as “normale” was considered to carry more of a value judgment. Besides this, the synthesized version was considered to be an adequate translation and adaptation of the original. The main resulting translation steps are provided in Supplementary File 1. The final version (AFQ-NL) can found in Supplementary File 2.

### Validity

Both the independent native Dutch speaker and the clinical expert approved of the final version.

**Fig. 1:**
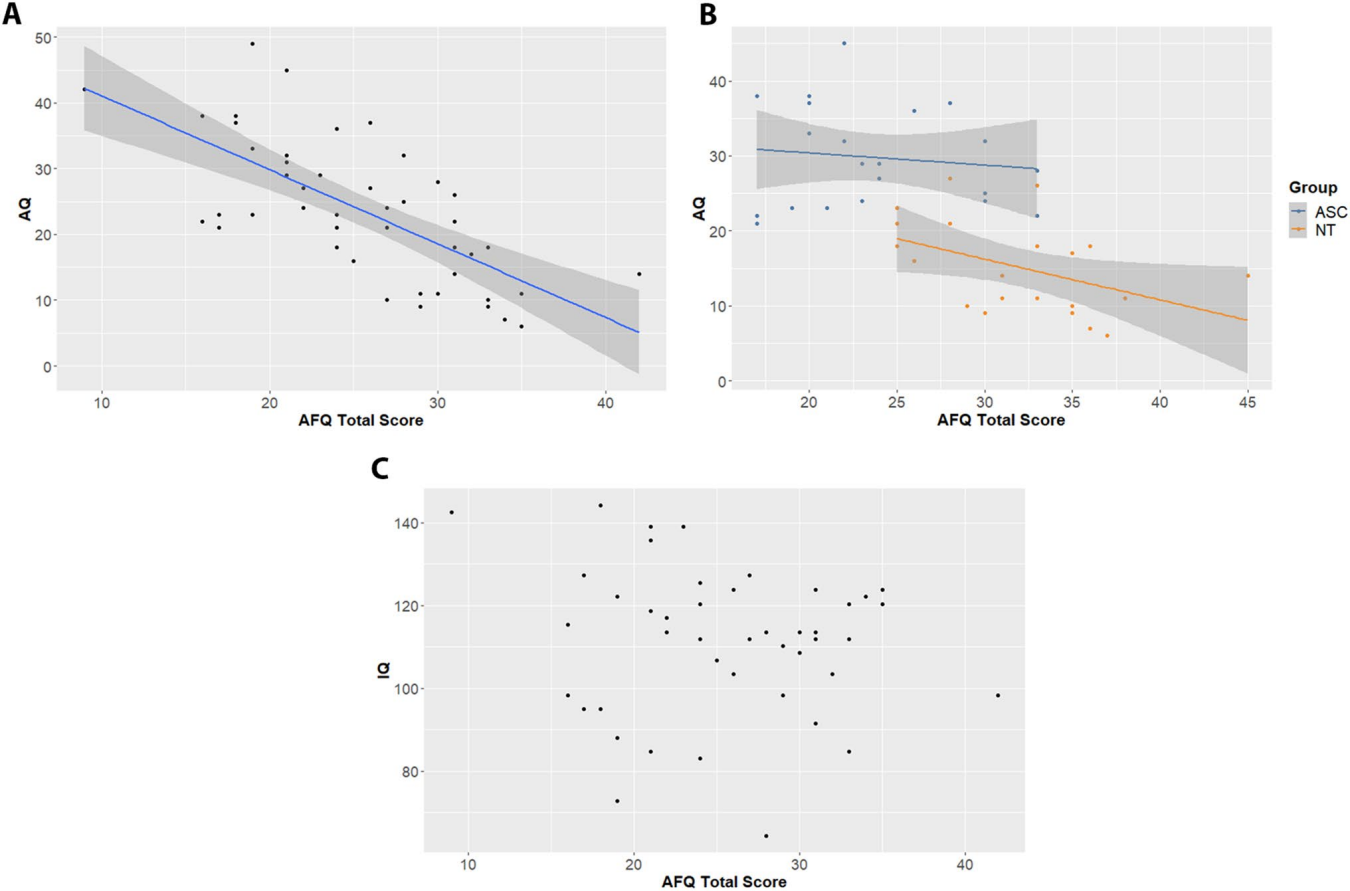
Convergent and divergent validity correlations. In panels **a** and **b**, AFQ score is given on the x-axis and AQ score is given on the y-axis. Panel **a** showed the correlation for the pooled sample (ASC and NT together), while panel **b** shows the separate fits for the two groups. In these panels, the blue line provides the linear fit, while the grey shaded area indicates the standard error. In panel **c**, IQ estimate is given on the y-axis. As no significant model fit was found, we do not include a fit line

For structural validity, we assessed convergent validity by testing whether the AFQ correlated with the AQ. As expected, we found a significant negative correlation (r = − 0.667, t = − 5.863, *p* < 0.001). However, when splitting this analysis by group, we found that this correlation was only present in the neurotypical group (r = − 0.454, t = − 2.223, *p* = 0.039), but not the ASC group (r = − 0.122, t = − 0.536, *p* = 0.598). See Fig. 1a. We assessed divergent validity by testing whether the AFQ correlated with estimated IQ. We found no evidence for such a correlation within our pooled sample, r = − 0.139, t = − 0.907, *p* = 0.369 (see Fig. 1b).

### Internal Consistency

Cronbach’s alpha for the total scale was 0.81, for the Feelings subscale 0.82, for the imagery subscale 0.27, and for the animation subscale 0.67.

## Discussion

The purpose of this study was to translate and cross-culturally adapt the Actions and Feelings Questionnaire (Williams et al., 2016) into Dutch and provide an assessment of the validity and internal consistency of this translation (AFQ-NL). Established guidelines for the translation and cross-cultural adaptation of self-report measures were followed (Beaton et al., 2000), resulting in a valid and reliable measurement tool.

The AFQ was designed in order to provide a short self-report measure of self-awareness of one’s own actions and the actions of others, and has been applied to both neurotypical (Williams et al., 2016) and ASC (Williams & Cameron, 2017) populations. Given the relevance and correlation of the AFQ to autistic traits (Huggins et al., 2019), we tested the convergent validity of the AFQ-NL in relation to AQ scores. We found, as expected, that higher AFQ scores correlate with lower AQ scores in the pooled sample. While this result was expected, it should be noted that the AFQ has previously only been linked to autistic traits in the general population, as well as empathy in ASC populations. Interestingly, we found that AFQ scores only correlated with autistic traits in the neurotypical group, and not in the ASC group. Contrary to the idea of empathy deficits in ASC (Baron-Cohen & Wheelwright, 2004), this fits well with the idea that empathy in ASC is quite heterogenous (Fletcher-Watson & Bird, 2020). As an additional test, we calculated divergent validity by checking whether there was a relationship between AFQ and IQ. As expected, we found no evidence for such a relationship. This finding demonstrates that the AFQ is not likely to be tapping into a cognitively complex skill set that is dependent upon IQ. Beyond divergent validity, this result therefore also suggests that the AFQ is not likely to be confounded by differences in IQ between or within study populations. These tests provide statistical evidence that the AFQ was successfully translated and adapted to the Dutch language.

The internal consistency of the total AFQ score was good (Cronbach’s alpha = 0.81), although two of the subscales (imagery and animation) showed lower alpha values. The animation subscale showed a Cronbach’s alpha of 0.67, which is just short of the recommended cut-off of 0.7. However, this is similar to the value presented by Williams and Cameron (2017). The somewhat lower value presented here may be explained by the relatively low sample size. However, it also suggests that this subscale is less consistent on its own. The imagery subscale showed a much lower alpha value of 0.27. This is also in line with Williams and Cameron (2017) who similarly showed reliability issues with the subset of items relating to imagery. Given that the internal consistency of the total AFQ score is quite high, and shows good validity, we suggest that the total score is a useful metric, although the subscales may be less reliable.

Inspecting the table of participant characteristics, we also see a strong correspondence with previous reports of the AFQ in neurotypical and ASC populations (Williams & Cameron, 2017). First, we replicate the finding of generally higher AFQ in neurotypical populations compared to ASC. Additionally, we see very similar values when comparing our study to that of Williams and Cameron (i.e., AFQ of 22–24 for ASC individuals in our study, compared to 20–23 in Williams and Cameron, and AFQ of 30–33 for NT individuals in our study, compared to 29–34 in Williams & Cameron, 2017). This provides additional evidence that the AFQ is not only successfully adapted for use in neurotypical populations, but also for use in ASC populations.

### Implications

Accumulating evidence suggests that many of the social and communicative difficulties experienced by ASC individuals are linked to the motor system (Cook, 2016), and thus to motor cognition. However, testing for motoric differences may require more specialized experimental and analytical techniques, which is not practical for clinicians. Availability of the AFQ in Dutch therefore provides clinicians in the Netherlands with an easy to collect assessment tool that can provide insights into one’s social-motor skills. Additionally, the AFQ can provide novel information for researchers, both for those working with ASC and those working with neurotypical populations. These insights can help to bridge our understanding of how the motor system, as an integrated aspect of human multimodal communication, relates to social functioning.

### Limitations

The main limitation of this study is the relatively small sample size when compared to many questionnaire-based studies. This is due to the fact that this validation and adaptation was part of a larger study involving behavioral and brain-imaging experiments, which limits the feasibility of obtaining large sample sizes. However, the COSMIN guideline for assessing content validity (Mokkink et al., 2020) suggests a minimum sample size of 50, while the guideline of Beaton et al. (2000) recommends a sample size of at least 30–40 participants. For both guidelines, our sample size is adequate. Additionally, while we showed convergent validity for the AFQ-NL in the neurotypical population, no correlation between AFQ and AQ was found in the ASC population. The originally reported correlation between AFQ and autistic traits was found in neurotypicals (Huggins et al., 2019) and we expected that correlation to extend to ASC individuals. However, AQ may not have been the best variable to calculate convergent validity for the ASC population. We are still confident, however, that the AFQ-NL is a valid adaptation also for ASC individuals, given the strong correspondence in AFQ scores between the current study and the values reported in Williams and Cameron (2017).

## Conclusion

The Actions and Feelings Questionnaire was successfully translated and cross-culturally adapted for use in the Netherlands, both for use in neurotypical and ASC populations.

## Supplementary Information

Below is the link to the electronic supplementary material.

Supplementary file1 (DOCX 20 kb)
