# Supplementary material for: Translation, Cross-Cultural Adaptation, and Validation of a Dutch Version of the Actions and Feelings Questionnaire in Autistic and Neurotypical Adults
Source: J Autism Dev Disord. 2021 May 18;52(4):1771–7. doi: 10.1007/s10803-021-05082-w (PMC8938389; doi:10.1007/s10803-021-05082-w)
Supplement: Supplementary file 1 — Supplementary file1 (DOCX 20 kb) [file 10803_2021_5082_MOESM1_ESM.docx]

**Supplementary File 1**

|  | **Original** | **Forward (Synthesis)** | **Backward** |
| --- | --- | --- | --- |
| 1 | I tend to pick up on people’s body language | *Ik ben in staat om iemand zijn of haar lichaamstaal te lezen* | I am able to read somebody’s body language. |
| 2 | To understand someone I rely on his or her words rather than their expression or gesture | *Om iemand te begrijpen, vertrouw ik meer op zijn of haar woorden dan de gebaren of uitdrukkingen van deze persoon* | In order to understand someone, I rely more on what they say than how they behave or carry themselves. |
| 3 | To make sense of what someone else is doing, I might copy his or her actions | *Om te begrijpen wat iemand anders doet, zal ik mogelijk zijn of haar acties kopiëren.* | In order to understand what somebody else does, I may copy their actions. |
| 4 | Music that I like makes me want to dance | *Wanneer ik muziek leuk vind, heb ik de neiging om te gaan dansen* | When I hear music that I like, I have the urge to dance. |
| 5 | In my mind’s eye, I often see myself doing things | *In mijn gedachten zie ik mijzelf vaak dingen doen.* | In my mind’s eye, I often see myself doing things. |
| 6 | If talking on the phone, I am sensitive to someone’s feelings by the tone of their voice | *Wanneer ik aan het bellen ben, ben ik gevoelig voor iemands gevoelens op basis van zijn of haar intonatie.* | When I am on the phone, I am sensitive to the other person’s feelings on the basis of their intonation. |
| 7 | If others are dancing I want to join in | *Als anderen aan het dansen zijn, wil ik meedoen* | If other people are dancing, I want to join them. |
| 8 | My body movements do not tend to reflect the way I feel | *Mijn lichaamsbewegingen reflecteren niet altijd hoe ik mij voel* | My body language does not always reflect how I feel. |
| 9 | I often imagine myself performing common actions | *Ik stel mijzelf regelmatig voor dat ik normale handelingen uitvoer.* | I regularly imagine that I perform normal actions. |
| 10 | I would consider myself to be a “touchy-feely” person | *Ik beschouw mijzelf als een gevoelig persoon* | I consider myself a sensitive person. |
| 11 | When I recall what someone said to me, I have to think hard to remember their facial expression at the time | *Wanneer ik terugdenk aan wat iemand tegen mij zei, kost het mij moeite om zijn of haar gezichtsuitdrukking voor de geest te halen* | When I think about what someone said to me, it costs a lot of effort to recall their facial expressions. |
| 12 | I rely on seeing how a person looks me in the eye to gauge what they really feel | *Ik beoordeel hoe iemand zich werkelijk voelt door de manier waarop diegene mij aankijkt* | I judge how someone feels based on how they look at me. |
| 13 | I wouldn’t tend to know what someone was feeling like if they did not say | *Ik zou niet goed kunnen inschatten wat mensen voelen als zij het niet zouden zeggen.* | I would not be able to estimate how someone feels if they do not say anything about it. |
| 14 | I move my hands a lot when I speak | *Ik beweeg mijn handen veel wanneer ik praat* | I move my hands a lot when I speak. |
| 15 | I get animated when I am enthusiastic in conversation | *Ik word levendig wanneer ik enthousiast ben in een gesprek* | I become animated when I am enthusiastic about a conversation. |
| 16 | I can easily bring to mind the look on someone’s face when I remember telling them something | *Als ik terugdenk aan het moment dat ik iemand iets vertelde, kan ik mij zijn of haar gezichtsuitdrukking gemakkelijk herinneren* | If I think back to a moment that someone told me something, I can easily recall their facial expressions. |
| 17 | Acting things out helps me to understand them | *Dingen uitbeelden helpt mij om zaken te begrijpen* | Acting things out helps me to understand things. |
| 18 | Watching someone’s body language is not a good way to judge their feelings | *Naar de lichaamstaal van een persoon kijken is niet een goede manier om iemands gevoelens in te schatten.* | Looking at body language is not a good way to estimate how someone is feeling. |

**Supplementary File 2**

***AFQ-NL***

1. *Ik ben in staat om iemands lichaamstaal te lezen*
2. *Om iemand te begrijpen vertrouw ik meer op diens woorden dan op de gebaren of uitdrukkingen van deze persoon*
3. *Om te begrijpen wat iemand anders doet, zal ik mogelijk diens acties kopiëren.*
4. *Wanneer ik muziek leuk vind, heb ik de neiging om te gaan dansen.*
5. *In mijn gedachten zie ik mezelf vaak dingen doen.*
6. *Wanneer ik met iemand aan het bellen ben, ben ik gevoelig voor diens gevoelens op basis van zijn of haar intonatie*
7. *Als anderen aan het dansen zijn, wil ik meedoen.*
8. *Mijn lichaamsbewegingen reflecteren niet altijd hoe ik mij voel.*
9. *Ik stel mijzelf regelmatig voor dat ik gewone handelingen uitvoer.*
10. *Ik beschouw mezelf als een gevoelig persoon.*
11. *Wanneer ik terugdenk aan wat iemand tegen mij zei, kost het mij moeite om diens gezichtsuitdrukking voor de geest te halen.*
12. *Ik beoordeel hoe iemand zich werkelijk voelt door de manier waarop diegene mij aankijkt.*
13. *Ik zou niet goed kunnen inschatten wat mensen voelen, wanneer zij dit niet zouden zeggen.*
14. *Ik beweeg mijn handen veel wanneer ik praat.*
15. *Ik word levendig wanneer ik enthousiast ben in een gesprek.*
16. *Als ik terugdenk aan het moment dat ik iemand iets vertelde, kan ik mij zijn of haar gezichtsuitdrukking gemakkelijk herinneren.*
17. *Dingen uitbeelden helpt mij om zaken te begrijpen.*
18. *Naar de lichaamstaal van een persoon kijken is niet een goede manier om iemands gevoelens in te schatten.*

***Instructions (NL)***

"Deze lijst bestaat uit een aantal uitspraken waarmee u het eens of oneens kunt zijn.

Lees iedere uitspraak zorgvuldig en kruis dan het antwoord aan dat het meest op u van toepassing is. Voor iedere uitspraak is een viertal antwoordmogelijkheden gegeven:

Helemaal mee eens
mee een
mee oneens
Helemaal mee oneens"

***Scoring***

Items are scored as 3 (*helemaal mee eens*), 2 (*mee eens*), 1 (*mee oneens*), 0 (*helemaal mee oneens*). Items 2, 8, 11, 13, and 18 should be reverse-scored.
